# Sirt3 Protects Cortical Neurons against Oxidative Stress via Regulating Mitochondrial Ca^2+^ and Mitochondrial Biogenesis

**DOI:** 10.3390/ijms150814591

**Published:** 2014-08-21

**Authors:** Shu-Hui Dai, Tao Chen, Yu-Hai Wang, Jie Zhu, Peng Luo, Wei Rao, Yue-Fan Yang, Zhou Fei, Xiao-Fan Jiang

**Affiliations:** 1 Department of Neurosurgery, Xijing Institute of Clinical Neuroscience, Xijing Hospital, Fourth Military Medical University, Xi’an 710032, China; E-Mails: fmmudaishuhui@163.com (S.-H.D.); fmmuchentao@163.com (T.C.); fmmuluopeng@163.com (P.L.); fmmuraowei@126.com (W.R.); fmmuyangyf@163.com (Y.-F.Y.); 2 Department of Neurosurgery, the 101th Hospital of People’s Liberation Army, Rescue Center of Craniocerebral Injuries of PLA, Wuxi 214044, China; E-Mails: wuxi101wyh@163.com (Y.-H.W.); wuxi101zhuj@163.com (J.Z.)

**Keywords:** Sirt3, oxidative stress, mitochondria, Ca^2+^, mitochondrial biogenesis

## Abstract

Oxidative stress is a well-established event in the pathology of several neurobiological diseases. Sirt3 is a nicotinamide adenine nucleotide (NAD^+^)-dependent protein deacetylase that regulates mitochondrial function and metabolism in response to caloric restriction and stress. This study aims to investigate the role of Sirt3 in H_2_O_2_ induced oxidative neuronal injury in primary cultured rat cortical neurons. We found that H_2_O_2_ treatment significantly increased the expression of Sirt3 in a time-dependent manner at both mRNA and protein levels. Knockdown of Sirt3 with a specific small interfering RNA (siRNA) exacerbated H_2_O_2_-induced neuronal injury, whereas overexpression of Sirt3 by lentivirus transfection inhibited H_2_O_2_-induced neuronal damage reduced the generation of reactive oxygen species (ROS), and increased the activities of endogenous antioxidant enzymes. In addition, the intra-mitochondrial Ca^2+^ overload, but not cytosolic Ca^2+^ increase after H_2_O_2_ treatment, was strongly attenuated after Sirt3 overexpression. Overexpression of Sirt3 also increased the content of mitochondrial DNA (mtDNA) and the expression of mitochondrial biogenesis related transcription factors. All these results suggest that Sirt3 acts as a prosurvival factor playing an essential role to protect cortical neurons under H_2_O_2_ induced oxidative stress, possibly through regulating mitochondrial Ca^2+^ homeostasis and mitochondrial biogenesis.

## 1. Introduction

Reactive oxygen species (ROS), including superoxide, hydroxyl radicals, and peroxides, are a group of molecules generated in the process of oxygen metabolism [1]. The endogenous stable oxidant hydrogen peroxide (H_2_O_2_) is considered as the principal ROS member, and has been widely used to induce oxidative stress in many different cell types [2]. In high concentrations, H_2_O_2_ results in cell injury by damaging key cellular molecules, such as DNA and lipids, and by inducing apoptosis, necrosis or autophagy [3,4]. H_2_O_2_ and other ROS-induced neuronal damage has been demonstrated to be involved in the etiology of several neurobiological disorders, ranging from acute insults, such as ischemic and traumatic brain injury to chronic neurodegenerative disorders, such as Alzheimer’s disease and Parkinson’s disease [5,6,7,8].

It has become increasingly clear that there are many complex signaling pathways involved in apoptosis after oxidative neuronal injury, including the intrinsic and extrinsic pathways. The intrinsic pathway is mediated by various stimuli that cause the release of cytochrome *c* from mitochondria into the cytoplasm [9,10]. In the cytoplasm, cytochrome *c* binds to the apoptosis protease activation factor (APAf-1) and forms a complex to induce the activation of pro-caspase 9 and initiate an enzymatic reaction cascade leading to the execution of apoptosis [11,12,13]. Several previous studies have demonstrated that many pharmacological agents and mitochondria associated molecules exert protective effects against neuronal injury through preservation of mitochondria function, and this might be an ideal neuroprotective strategy [14,15].

The sirtuins (or Sir2-like proteins) are a conserved family of class III histone deacetylases (HDACs), and have been reported to be involved in transcriptional silencing, genetic control of aging and longevity of organisms ranging from yeasts to humans [16]. Among the known sirtuin members, Sirt3 is characterized by its localization to the mitochondria, and has been identified as a stress responsive deacetylase recently shown to play a role in protecting cells under stress conditions [17,18,19]. Mitochondrial Sirt3 was shown to act as a pro-survival factor playing an essential role to protect neurons under excitotoxicity [20]. A recent report also showed that Sirt3-mediated deacetylation of FOXO3 attenuates oxidative stress induced mitochondrial dysfunction via the coordination of mitochondrial biogenesis, fission/fusion and mitophagy [21]. However, the clear role of Sirt3 in oxidative stress-induced neuronal injury has not been previously reported. Therefore, the aim of the present study is to investigate the role of Sirt3 in H_2_O_2_-induced neuronal injury in primary cultured cortical neurons, as well as the potential mechanisms with focus on mitochondrial calcium metabolism and mitochondrial biogenesis.

## 2. Results

### 2.1. Expression of Sirt3 after H_2_O_2_ Treatment in Cortical Neurons

**Figure 1:**
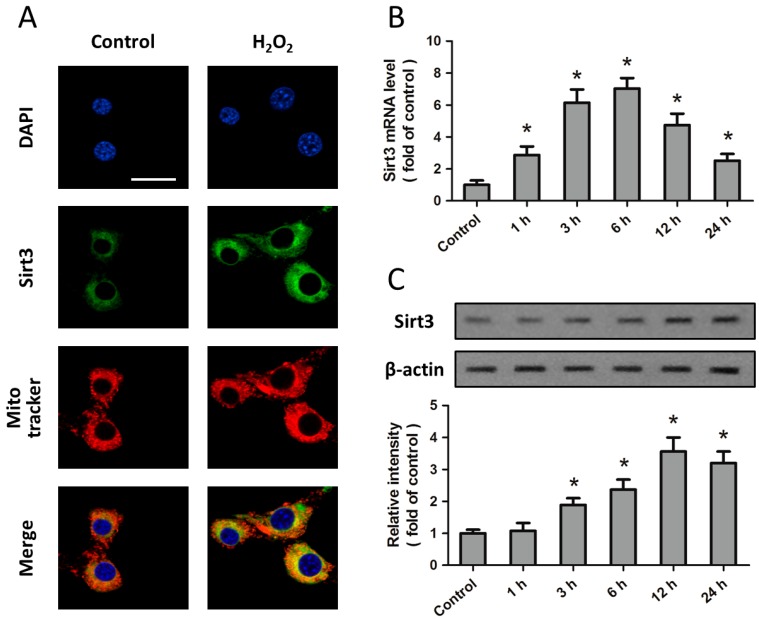
Expression of Sirt3 after H_2_O_2_ treatment in cortical neurons. Cortical neurons were treated with 0.1 mM H_2_O_2_ for 24 h, and the expression and distribution of Sirt3 was detected by immunofluorescence staining for Sirt3 (green), Merge (yellow); mitochondria (red) and DAPI (blue) (**A**); The expression of *sirt3* mRNA (**B**) and protein (**C**) was measured by Real-Time RT-PCR and Western blot, respectively. Scale bar: 50 μm. Data are shown as mean ± SD of five experiments. * *p* < 0.05 *vs.* Control.

Expression of Sirt3 was examined in primary cultured cortical neurons to test their feasibility in studying the biological function of Sirt3 in oxidative stress. Immunostaining results showed that Sirt3 is localized in the cytoplasma, but not in the nucleus, which was counterstained with DAPI (Figure 1A). To investigate the effect of oxidative stress on Sirt3 expression, neurons were treated with H_2_O_2_ (0.1 mM) for 24 h, and the expression of *sirt3* mRNA and protein was detected by RT-PCR or Western blot at different time points (control, 1, 3, 6, 12, and 24 h). The levels of *sirt3* mRNA and protein were both significantly increased within 24 h of the start of H_2_O_2_ treatment, and peaked at 6 or 12 h, respectively (Figure 1B,C). In addition, the distribution of Sirt3 was unaffected by H_2_O_2_ treatment (Figure 1A).

### 2.2. H_2_O_2_-Induced Sirt3 Expression Promotes Neuronal Survival

**Figure 2:**
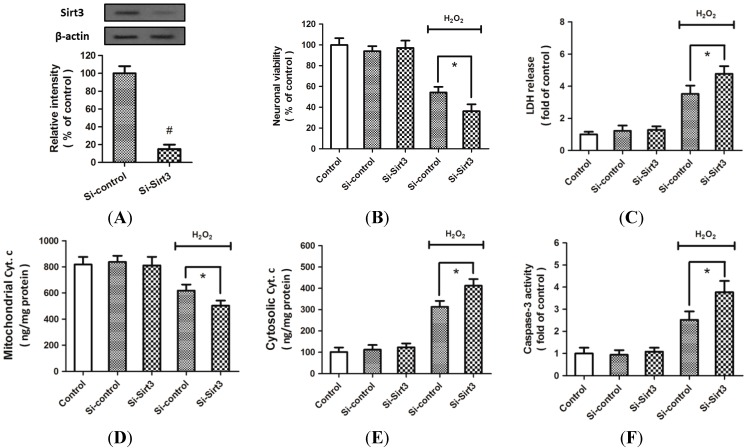
Knockdown of Sirt3 aggravates H_2_O_2_-induced neurotoxicity. Cortical neurons were transfected with Sirt3-specific siRNA (Si-Sirt3) or control siRNA (Si-control) for 72 h. The expression of Sirt3 was measured by Western blot analysis (**A**); After transfection, neurons were treated with 0.1 mM H_2_O_2_ for 24 h to induce oxidative stress. The neuronal viability was measured by WST-1 assay (**B**); and cytotoxicity was measured by LDH assay (**C**); The mitochondrial cytochrome *c* (**D**); cytosolic cytochrome *c* (**E**); and caspase-3 activity (**F**) were measured. Data are shown as mean ± SD of five experiments. ^#^ *p* < 0.05 *vs.* Si-control. * *p* < 0.05 *vs.* Si-control.

To investigate the biological functions of Sirt3 in H_2_O_2_-induced neurotoxicity, cortical neurons were transfected with Sirt3 specific siRNA (Si-Sirt3) or control siRNA (Si-control). Western blot analysis indicated that Sirt3 expression was significantly reduced in neurons after their transfection with Si-Sirt3 (Figure 2A). After treatment with 0.1 mM H_2_O_2_ for 24 h, the viability of the neurons transfected with Si-Sirt3 was lower than that of neurons transfected with Si-control (Figure 2B), whereas the lactate dehydrogenase (LDH) release in Si-Sirt3 transfected neurons was higher than that in cells transfected with Si-control (Figure 2C). To investigate the effects of Sirt3 in neuronal apoptosis, we also measured the levels of cytochrome *c* in mitochondria and cytoplasma, and caspase-3 activity after H_2_O_2_ treatment. The results showed that knockdown of Sirt3 significantly increased the release of cytochrome *c* from mitochondria into cytoplasma (Figure 2D,E), and further increased the activation of caspase-3 induced by H_2_O_2_ (Figure 2F).

### 2.3. Overexpression of Sirt3 Reduces H_2_O_2_-Induced Neuronal Injury

**Figure 3:**
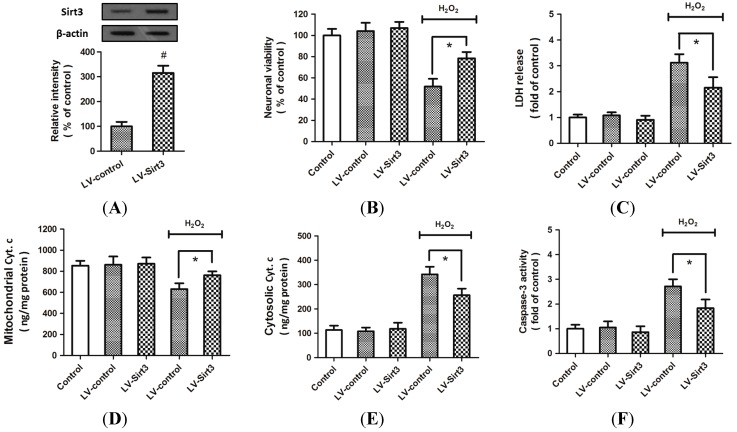
Overexpression of Sirt3 reduces H_2_O_2_-induced neuronal injury. Cortical neurons were transfected with lentivirus expressed Sirt3 (LV-Sirt3) or control lentivirus (LV-control) for 72 h, and the expression of Sirt3 was measured by Western blot analysis (**A**); After transfection, HT22 cells were treated with or without 0.1 mM H_2_O_2_ for 24 h to induce oxidative stress. The neuronal viability was measured by WST-1 assay (**B**); and cytotoxicity was measured by LDH assay (**C**); The mitochondrial cytochrome *c* (**D**); cytosolic cytochrome *c* (**E**); and caspase-3 activity (**F**) were measured. Data are shown as mean ± SD of five experiments. ^#^ *p* < 0.05 *vs.* LV-control. * *p* < 0.05 *vs.* LV-control.

To confirm the protective effects of Sirt3 on H_2_O_2_-induced neuronal injury, cortical neurons were transfected with lentivirus expressed Sirt3 (LV-Sirt3) or control lentivirus (LV-control). The results of Western blot analysis indicated that the expression of Sirt3 was significantly increased by LV-Sirt3 transfection as compared to LV-control (Figure 3A). The decreased neuronal viability (Figure 3B) and the increased LDH release (Figure 3C) induced by H_2_O_2_ treatment were all partially reversed by Sirt3 overexpression. As shown in Figure 3D,E, overexpression of Sirt3 significantly attenuated the cytochrome *c* release from mitochondria into cytoplasma. A similar result in caspase-3 activity was also observed (Figure 3F). All these data suggested that overexpression of Sirt3 protected cortical neurons form H_2_O_2_-induced neuronal injury through inhibiting apoptotic cell death.

### 2.4. Overexpression of Sirt3 Inhibits H_2_O_2_-Induced Oxidative Stress

**Figure 4:**
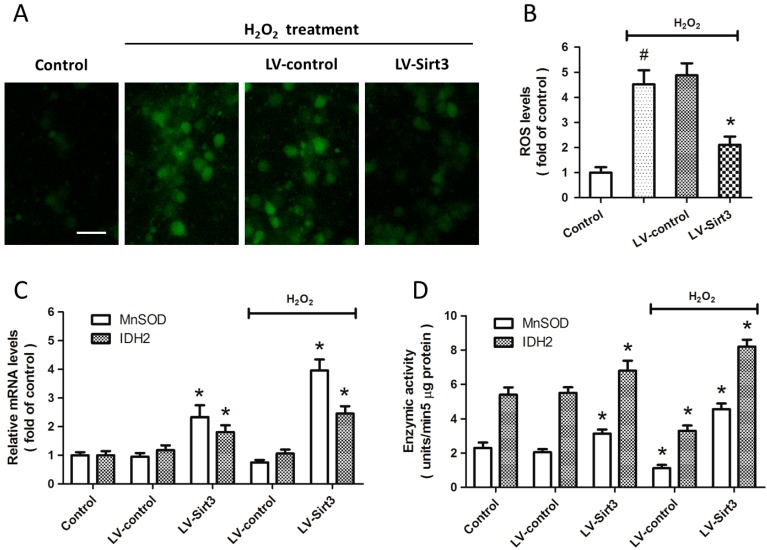
Overexpression of Sirt3 inhibits H_2_O_2_-induced oxidative stress. Cortical neurons were transfected with lentivirus expressed Sirt3 (LV-Sirt3) or control lentivirus (LV-control) for 72 h, and then treated with or without 0.1 mM H_2_O_2_. The ROS production was measured by H_2_DCFDA staining (**A**) and calculated (**B**); The mRNA levels of MnSOD and IDH2 were measured by Real-Time RT-PCR (**C**); The enzymatic activity of MnSOD and IDH2 was assayed (**D**); Scale bar: 50 μm. Data are shown as mean ± SD of five experiments. ^#^ *p* < 0.05 *vs.* Control. * *p* < 0.05 *vs.* LV-control.

To determine whether Sirt3 affects the generation of intracellular ROS, cortical neurons were transfected with lentivirus expressed Sirt3 (LV-Sirt3) or control lentivirus (LV-control) for 72 h, and the ROS production was measured by H_2_DCFDA staining (Figure 4A). The results showed that H_2_O_2_ treatment significantly increased the ROS generation, whereas Sirt3 overexpression attenuated the intracellular ROS levels compared with LV-control transfected group (Figure 4B). We also measured the expression and enzymatic activities of MnSOD and IDH2, two important endogenous anti-oxidative enzymes in H_2_O_2_ related neuronal injury. The results of Real-Time RT-PCR showed that the mRNA levels of MnSOD and IDH2 were both significantly increased by Sirt3 overexpression, even in the absence of H_2_O_2_ treatment (Figure 4C). As shown in Figure 4D, a similar result on enzymatic activity of these two enzymes was also observed.

### 2.5. Overexpression of Sirt3 Blocks H_2_O_2_-Induced Mitochondrial Ca^2+^ Dysregulation

**Figure 5:**
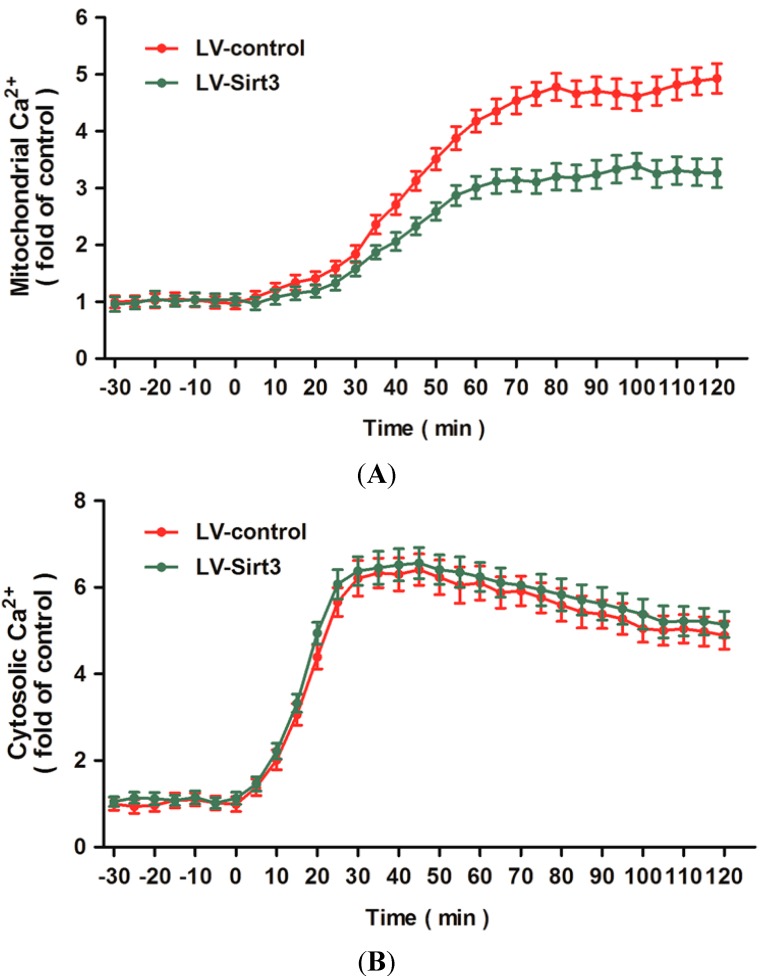
Overexpression of Sirt3 blocks H_2_O_2_-induced mitochondrial Ca^2+^ dysregulation. After transfection with lentivirus expressed Sirt3 (LV-Sirt3) or control lentivirus (LV-control) for 72 h, cortical neurons were treated with 0.1 mM H_2_O_2_. The mitochondrial Ca^2+^ concentration (**A**) and cytosolic Ca^2+^ concentration (**B**) were measured up to 120 min after H_2_O_2_ treatment. Data are shown as mean ± SD of five experiments.

To characterize the effects of Sirt3 on intracellular calcium homeostasis, we used calcium imaging to detect the changes of mitochondrial and cytosolic Ca^2+^ concentrations after H_2_O_2_ treatment. Figure 5A shows dynamic changes of mitochondrial Ca^2+^ as monitored by Rhod-2 AM probe and expressed as fold of the baseline for up to 120 min following H_2_O_2_ treatment. Overexpression of Sirt3 attenuated the increase of mitochondrial Ca^2+^ induced by H_2_O_2_ treatment from 30 to 120 min after neurotoxicity. In addition, we also measured the changes of cytosolic Ca^2+^ after H_2_O_2_ treatment with or without Sirt3 overexpression (Figure 5B). The results showed that H_2_O_2_ triggered a rapid rise in cytosolic Ca^2+^ within 10 min that slowly decreased from 40 to 120 min after injury. Overexpression of Sirt3 had no effects on the changes of cytosolic Ca^2+^ after H_2_O_2_ treatment within 120 min.

### 2.6. Overexpression of Sirt3 Promotes Mitochondrial Biogenesis after H_2_O_2_ Treatment

**Figure 6:**
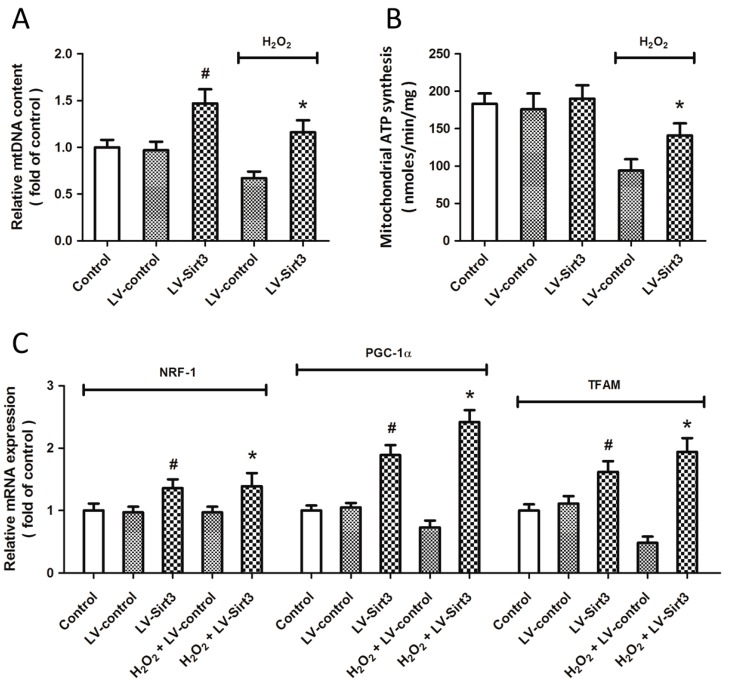
Overexpression of Sirt3 promotes mitochondrial biogenesis after H_2_O_2_ treatment. After transfection with lentivirus expressed Sirt3 (LV-Sirt3) or control lentivirus (LV-control) for 72 h, cortical neurons were treated with 0.1 mM H_2_O_2_. The mitochondrial DNA (mtDNA) content (**A**) and mitochondrial ATP generation (**B**) were measured. The expression of NRF-1, PGC-1α and TFAM at mRNA levels was measured by Real-Time RT-PCR (**C**). Data are shown as mean ± SD of five experiments. ^#^ *p* < 0.05 *vs.* Control. * *p* < 0.05 *vs.* LV-control.

To determine whether Sirt3 affects the mitochondrial biogenesis, cortical neurons were transfected with lentivirus expressed Sirt3 (LV-Sirt3) or control lentivirus (LV-control) for 72 h before H_2_O_2_ treatment. The content of mitochondrial DNA (mtDNA) was measured (Figure 6A), and the results showed that H_2_O_2_ treatment significantly decreased mtDNA content, whereas Sirt3 overexpression increased mtDNA content both in the present and absence of H_2_O_2_ treatment. The mitochondrial ATP synthesis was also preserved by LV-Sirt3 transfection compared with that in LV-control transfected neurons after injury (Figure 6B). In addition, we also measured the expression of NRF-1, PGC-1α, and transcription factor A, mitochondrial (TFAM), three mitochondrial biogenesis factors, using Real-Time RT-PCR (Figure 6C). The results showed that Sirt3 overexpression significantly increased the mRNA levels of these factors both before and after H_2_O_2_ treatment.

## 3. Discussion

Mammalian sirtuin members were originally identified as histone deacetylases that are associated with numerous physiological roles, such as stress response, regulation of metabolism, gene silencing, and aging [22,23]. Recently, emerging evidence suggests that sirtuins are dynamically regulated at both transcriptive and translational levels under pathological conditions and might play important roles in various human diseases. For example, increased expression of Sirt1 was shown to be involved in the regulation of neuronal survival and death by deacetylation of molecules such as p53 and PGC-1α [24,25]. Increased expression of Sirt3 was observed in hearts under stress conditions, whereas Sirt3 levels were reduced in hypertrophied or failing hearts [18,26]. Sirt3 is the mitochondrial matrix protein that is present in high amount in mouse kidney, heart, liver, and brain tissues, which are all organs with high ATP demands [27,28]. Importantly, neuronal Sirt3 was shown to be up-regulated after *N*-methyl-d-aspartic acid (NMDA) treatment and protects cortical neurons against excitotoxic injury through inhibiting NAD depletion and subsequent oxidative stress [20]. In the present study, we found that the expression of Sirt3 was increased after H_2_O_2_ treatment in a time-dependent manner in primary cultured cortical neurons. Knockdown of Sirt3 by specific siRNA transfection aggravated H_2_O_2_ induced neuronal injury, and overexpression of Sirt3 exerted neuroprotective effects. These data indicated that Sirt3 may be an endogenous protective mechanism under oxidative stress, and overexpression of Sirt3 would provide an incremental protective effect against H_2_O_2_ injury.

The mitochondrion plays a prominent role in the induction of apoptotic cell death after oxidative stress [29]. It is well-known that acetylation of mitochondrial proteins may play a role in maintaining and regulating mitochondrial metabolism and function [28]. Sirt3 is localized in mitochondria and responds to changes in cellular and nutrient stress resulting in the activation of their deacetylase or ribosyltransferase activity resulting in a post-translational modification of downstream target proteins that direct intracellular oxidation/reduction pathways [30,31]. However, the exact role of Sirt3 in apoptosis is contradictory, largely depending upon cell types. Previous studies have demonstrated the pro-apoptotic effects of Sirt3 in leucocythemia and colorectal cancer cells [32,33], whereas the anti-apoptotic activity of Sirt3 and Sirt3-mediated anti-apoptotic mechanism were reported by several other studies [20,34,35]. Recent observations also hint at additional neuroprotective effects of Sirt3 involving regulation of mitochondrial dynamics [17,36,37]. In the present study, we found that Sirt3 inhibited the activation of caspase-3 and cytochrome *c* release from mitochondria into cytoplasma after H_2_O_2_ treatment in cortical neurons, indicating the anti-apoptotic activity of Sirt3 in oxidative neuronal injury. In consistent with our findings, a previous study showed that Sirt3 is required for the regulation of cytochrome *c* superoxide-scavenging capacity via deacetylation and activation of complex IV [38]. In addition, under caloric restriction (CR) conditions, upregulated expression of Sirt3 directly deacetylated cyclophilin D (CypD), preventing its association with adenine nucleotide translocator (ANT) and, therefore, blocking mPTP formation [39,40]. All these data indicated that the protective effects of Sirt3 against oxidative stress in neurons could partially be mediated by mitochondrial associated apoptosis. Thus, we speculate that Sirt3 might exert anti-apoptotic activity under various pathological conditions except for in cancer cells, which needs to be further investigated.

One of the key events that cause mitochondria related apoptosis is an abnormal increase in intracellular Ca^2+^, induced by receptor-mediated Ca^2+^ influx or Ca^2+^ release from intracellular stores, and followed mitochondrial Ca^2+^ overload [41,42]. Intra-mitochondrial Ca^2+^ uptake seems to play a two-faced role in the regulation of mitochondrial function: be beneficial for mitochondrial function in the processes of oxidation phosphorylation and ATP synthesis, or be detrimental in instigating subsequent pathological cascades [43]. Recent evidence have validated mitochondrial Ca^2+^ overload as a key effector in the apoptotic response induced by stimuli like oxygen and glucose deprivation (OGD), H_2_O_2_, or arachidonic acid [44,45]. In consistent with these findings, we found in the present study that H_2_O_2_ treatment significantly increased the intracellular and intra-mitochondrial Ca^2+^ concentrations, which confirmed the involvement of intracellular Ca^2+^ overload and mitochondrial Ca^2+^ uptake in our *in vitro* neuronal injury model. The mechanism underlying mitochondrial Ca^2+^ loading in apoptotic cell death under oxidative stress is associated with nitric oxide production, cytochrome *c* dissociation, mitochondrial permeability transition pore (mPTP) opening with release of cytochrome *c*, and Ca^2+^-calmodulin dependent protein kinases activation [46]. The anti-apoptotic Bcl-2 was shown to reduce mitochondrial Ca^2+^ uptake by decreasing the releasable endocytoplasmic reticulum (ER) Ca^2+^ pool, whereas the ER resident fraction of Bax amplified Ca^2+^-mediated cell death [47,48,49]. Inhibition of Ca^2+^ transport into mitochondria through targeting the mitochondrial calcium uniporters (mCU), such as uncoupling proteins (UCPs), has been demonstrated to protect neurons from cell death mediated by glutamate [50,51,52]. Here, we found that overexpression of Sirt3 significantly reduced mitochondrial Ca^2+^ uptake after H_2_O_2_ treatment. Intriguingly, the increased intracellular Ca^2+^ concentration after oxidative injury was not altered by Sirt3 overexpression compared with that in LV-control transfected neurons. Intracellular Ca^2+^ concentration is differently regulated by Ca^2+^ influx from extracellular spaces, Ca^2+^ release from the ER stores and mitochondrial Ca^2+^ uptake, as well as the interaction between these factors [46,53]. Thus, we speculate that Sirt3 is involved in the Ca^2+^ transfer from cytoplasm to mitochondria through the regulation of mCU without effects on the Ca^2+^ influx from extracellular spaces, or the intra-mitochondrial Ca^2+^ overload was due to the Ca^2+^ influx from the ER stores into the mitochondria, which has been demonstrated in ischemic neuronal injury models [54].

In our study, a decline in intracellular ROS generation after Sirt3 overexpression was accompanied by increased mRNA levels and enzymatic activities of MnSOD and IDH2. Increased activity of endogenous antioxidant enzymes, an important marker to assess mitochondrial function, can be the result of increased mitochondrial biogenesis, which is defined as the growth and division of mitochondria [55]. Mitochondrial homeostasis maintains mitochondrial function and integrity through the generation of new mitochondria and the selective degradation of defective mitochondria, the most important function of mitochondrial biogenesis [56]. Mitochondrial biogenesis has been found to be helpful for attenuating any detrimental consequence of oxidative stress and has been suggested as a novel component of the repair mechanism in central nervous system (CNS) [55,57]. We detected the total amount of intact mtDNA and the mRNA levels of NRF-1, PGC-1α and TFAM, three mitochondrial-specific transcription factors [58,59], and significant decreases of these factors were found after H_2_O_2_ treatment, indicating damaged mitochondrial biogenesis function in oxidative stress conditions. The capacity for mitochondrial biogenesis decreases with increasing age [56], and Sirt3 has been shown to be a key regulator in aging and longevity of organisms, suggesting that Sirt3 might be a regulator of mitochondrial biogenesis during oxidative. Importantly, caloric restriction (CR)-induced activation of Sirt1 was shown to promote mitochondrial biogenesis via increases in PGC-1α, NRF-1 and TFAM [60]. In the present study, overexpression of Sirt3 significantly increased mtDNA content and the three transcript factors both in the presence and absence of H_2_O_2_ insult, which was also demonstrated in recent studies [21]. All these data indicate that Sirt3-induced neuroprotection against oxidative stress is partially mediated by enhancement of mitochondrial biogenesis.

There are some limitations to our study. First, Sirt3 is shown to be expressed in both neurons and glial cells [61], which play important roles in the regulation of neuronal survival and/or death under oxidative stress conditions, and only cultured cortical neurons were studied in the present study. Thus, it needs to be further investigated that whether the conclusion in the present study can also apply to glial cells or neuron-glia co-cultures. In addition, although mitochondrial Ca^2+^ uptake is considered as an important regulator of intracellular Ca^2+^ homeostasis, it is well-known that the ER Ca^2+^ stores may also be pivotal for Ca^2+^ overload associated neuronal cell death [62,63]. Several ER related proteins, such as glucose-regulated protein 78 (GRP78) and C/EBP homologous protein (CHOP), can be regulated by histone deacetylase inhibitors [64,65]. Thus, the ER related Ca^2+^ homeostasis and its interaction with mitochondrial proteins need more extensive study after Sirt3 knockdown or overexpression.

## 4. Experimental Section

### 4.1. Primary Culture of Cortical Neurons

Cortical neurons were cultured from Sprague–Dawley rats using a modified method reported by Redmond *et al*. [66]. Briefly, cerebral cortices were removed from embryos at 16–18 days, stripped of meninges and blood vessels and minced. Tissues were dissociated by 0.25% trypsin digestion for 15 min at 37 °C and gentle trituration. Neurons were resuspended in Neurobasal medium containing 2% B27 supplement and 0.5 mM l-Glutamine and plated at a density of 3 × 10^5^ cells/cm^2^. Before seeding, culture vessels, consisting of 96-well plates, 1.5 cm glass slides or 6 cm dishes were coated with poly-l-lysine (PLL, 50 μg/mL) at room temperature overnight. Neurons were maintained at 37 °C in a humidified 5% CO_2_ incubator and half of the culture medium was changed every other day.

### 4.2. Immunocytochemistry

Immunocytochemistry was used to detect the expression of Sirt3 in cortical neurons. Briefly, after being fixed with 4% paraformaldehyde for 15 min at room temperature, neurons were permeabilized with 0.2% Triton X-100, and incubated with primary antibody (anti-Sirt3, 1:100, Santa Cruz, CA, USA) overnight at 4 °C. The neurons were then incubated with the Alexa-488-conjugated mouse-anti-rabbit secondary antibody (Invitrogen, Carlsbad, CA, USA, 1:500) for 2 h at 37 °C. 4,6-diamidino-2-phenylindole (DAPI, 10 μg/mL, Santa Cruz, CA, USA) was used to stain nucleus. Mito-tracker (Invitrogen, Carlsbad, CA, USA, 1:1000) was used to stain the mitochondria. Images were captured with an Olympus FV10i Confocal Microscope (Tokyo, Japan).

### 4.3. Short Interfering RNA (siRNA) and Transfection

The specific siRNA targeted Sirt3 (Si-Sirt3, sc-61556, Santa Cruz, CA, USA) and control siRNA (Si-control, sc-37007, Santa Cruz, CA, USA), which should not knock down any known proteins, were purchased from Santa Cruz Biotechnology, Inc. (Santa Cruz, CA, USA). The above siRNA molecules were transfected with Lipofectamine 2000 (Invitrogen, Carlsbad, CA, USA) in 6-well plates for 72 h. After transfection, the cortical neurons were treated with H_2_O_2_ (0.1 mM) for 24 h and subjected to various measurements.

### 4.4. Lentivirus Construction and Transfection

The coding sequence of Sirt3 was amplified by RT-PCR. The primer sequences were: forward, 5'-TACTTCCTTCGGCTGCTTCA-3'; reverse, 5'-AAGGCGAAATCAGCCACA-3'. The PCR fragments and the pGC-FU plasmid (GeneChem, Shanghai, China) were digested with Age I and then ligated with T4 DNA ligase to produce pGC-FU-Sirt3. To generate the recombinant Lentivirus LV-Sirt3, 293T cells were co-transfected with the pGC-FU plasmid (20 μg) with a cDNA encoding Sirt3, pHelper1.0 plasmid (15 μg) and pHelper 2.0 plasmid (10 μg) by using Lipofectamine 2000 (100 μL). The supernatant was harvested and the viral titer was calculated by transducing 293T cells. As a control, we also generated a control lentiviral vector that expresses GFP alone (LV-control). Cortical neurons were transfected with lentivirus vectors for 72 h and subjected to various treatments.

### 4.5. Neuronal Viability Assay

Neuronal viability assay was performed by using the Cell Proliferation Reagent WST-1 following the manufacture’s protocol (Roche, Basel, Switzerland). Briefly, cortical neurons were cultured at a concentration of 5 × 10^4^ in microplates in a final volume of 100 μL/well culture medium. After various treatments, 10 μL cell proliferation reagent, WST-1, was added into each well and incubated for 4 h at 37 °C. Then, 100 μL/well culture medium and 10 μL WST-1 was added into one well in the absence of neurons, and its absorbance was used as a blank position for the ELISA reader. Cells were shaken thoroughly for 1 min on a shaker and the absorbance of the samples was measured using a microplate reader.

### 4.6. Lactate Dehydrogenase (LDH) Release Assay

Cytotoxicity was determined by the release of LDH, a cytoplasmic enzyme released from cells. LDH release into the culture medium was detected using a diagnostic kit according to the manufacturer’s instructions (Jiancheng Bioengineering Institute, Nanjing, China). Briefly, 50 µL of supernatant from each well was collected to assay LDH release. The samples were incubated with reduced form of nicotinamide-adenine dinucleotid (NADH) and pyruvate for 15 min at 37 °C and the reaction was stopped by adding 0.4 M NaOH. The activity of LDH was calculated from the absorbance at 440 nm and background absorbance from culture medium that was not used for any cell cultures was subtracted from all absorbance measurements. The results were presented as the fold of control.

### 4.7. Quantification of Cytochrome c Release

Cytochrome *c* release into the cytoplasm was assessed after subcellular fraction preparation. Cortical neurons were washed with ice-cold phosphate buffered sodium (PBS) for three times and lysed with a lysis buffer containing protease inhibitors. The cell lysate was centrifuged for 10 min at 750× *g* at 4 °C, and the pellets containing the nuclei and unbroken cells were discarded. The supernatant was then centrifuged at 15,000× *g* for 15 min. The resulting supernatant was removed and used as the cytosolic fraction. The pellet fraction containing mitochondria was further incubated with PBS containing 0.5% Trition X-100 for 10 min at 4 °C. After centrifugation at 16,000× *g* for 10 min, the supernatant was collected as mitochondrial fraction. The levels of cytochrome *c* in cytosolic and mitochondrial fractions were measured using the Quantikine M Cytochrome *c* Immunoassay kit obtained from R&D Systems (R&D, Minneapolis, MN, USA). Data were expressed as ng/mg protein.

### 4.8. Measurement of Caspase-3 Activity

The activity of caspase-3 was measured using the colorimetric assay kit according to the manufacturer’s instructions (Cell Signaling Technology, Beverly, MA, USA). Briefly, after being harvested and lysed 10^6^ cells were mixed with 32 μL of assay buffer and 2 μL of 10 mM Ac-DEVD-pNA substrate. Absorbance at 405 nm was measured after incubation at 37 °C for 4 h. Absorbance of each sample was determined by subtraction of the mean absorbance of the blank and corrected by the protein concentration of the cell lysate. The results were described as relative activity to that of control group.

### 4.9. Measurement of ROS Generation

Briefly, cortical neurons were incubated with 2,7-dichlorofluorescein diacetate (DCF-DA) (Sigma, St. Louis, MO, USA) (10 μM) for 1 h at 37 °C in the dark, and then resuspended in PBS. Intracellular ROS production was detected using the fluorescence intensity of the oxidant-sensitive probe 2,7-dichlorodihydro-fluorescein diacetate (H_2_DCF-DA) in a microscope and the fluorescence was read using an excitation wavelength of 480 nm and an emission wavelength of 530 nm.

### 4.10. Detection of Antioxidant Enzymes Activities

The enzymatic activity of manganese superoxide dismutase (MnSOD) and isocitrate dehydrogenase 2 (IDH2) was measured by use of a commercially available assay kits following the manufacturer’s instruction (Cayman Chemical, Ann Arbor, MI, USA). Protein concentration was determined by using BCA protein kit (Jiancheng Bioengineering Institute, Nanjing, China). The enzyme activities were then normalized to the corresponding protein concentration for each sample.

### 4.11. Calcium Imaging

Intracellular calcium concentrations were measured by Fura-2-AM [67]. Cortical neurons grown on glass slides were loaded with 5 μM Fura-2-AM for 30 min before H_2_O_2_ treatment at room temperature. Cells were then placed in an open-bath imaging chamber containing Dulbecco’s NaCl/P_i_ (Invitrogen, Carlsbad, CA, USA) supplemented with 20 mM glucose. With a Nikon inverted fluorescence microscope (Tokyo, Japan), cells were excited at 345 and 385 nm, and the emission fluorescence at 510 nm was recorded. Images were collected and analyzed with METAFLUOR image-processing software. The Ca^2+^ concentration values were then calculated, and Ca^2+^-insensitive fluorescence was subtracted from each wavelength before calculations were performed. Cell permeable Rhod-2 AM was used as a mitochondria selective Ca^2+^ indicator. The cells were loaded with 2 mM Rhod-2 AM for 30 min before various treatments, and then washed three times. The fluorescence intensities were immediately analyzed with a Nikon inverted fluorescence microscope. Results are presented as the fold of the control.

### 4.12. Determination of Mitochondrial DNA Content

Long fragment PCR was used to quantify the relative abundance of intact mtDNA as previously described [68]. As an internal standard, rat DNA derived from normal rat cortical neurons was added to the PCR reaction mixture for each sample. The primers used for the amplification of 14.3 kbp mitochondrial genomes for rat were: forward, 5'-ATATTTTCACTGCTGAGTCCCGTGG-3'; reverse, 5'-AATTTCGGTTGGGGTGACCTCGGAG-3'. Band densitometry was semi-quantitatively analyzed using Image J software (National Institutes of Health, Boston, MA, USA).

### 4.13. Measurement of ATP Synthesis

Isolated mitochondria were utilized to measure ATP synthesis with a luciferase/luciferin-based system as described elsewhere [69]. Thirty micrograms of mitochondria- enriched pellets were resuspended in 100 μL of buffer A (150 mM KCl, 25 mM Tris-HCl, 2 mM potassium phosphate, 0.1 mM MgCl_2_, pH 7.4) with 0.1% BSA, 1 mM malate, 1 mM glutamate, and buffer B (containing 0.8 mM luciferin and 20 mg/mL luciferase in 0.5 M Tris-acetate pH 7.75). The reaction was initiated by addition of 0.1 mM ADP and monitored for 5 min using a microplate reader.

### 4.14. Real-Time RT-PCR

Total RNA was isolated from cortical neurons using Trizol reagent (Invitrogen, Carlsbad, CA, USA). After the equalization of the RNA quantity in each group, the mRNA levels were quantitated using a Bio-Rad iQ5 Gradient Real-Time PCR system (Bio-Rad Laboratories, Richmond, CA, USA), and GAPDH was used as an endogenous control. Primers for all Real-Time PCR experiments were listed as follow: Sirt3: forward: 5'-TACTTCCTTCGGCTGCTTCA-3', reverse: 5'-AAGGCGAAATCAGCCACA-3'; MnSOD: forward: 5'-TTAACGCGCAGATCATGCA-3', reverse: 5'-GGTGGCGTTGAGATTGTTCA-3'; IDH2: forward: 5'-AATTTTAGGACCCCCGTCTG-3', reverse: 5'-GGGGTGAAGACCATTTTGAA-3'; NRF-1: forward: 5'-GAGTGACCCAAACCGAACA-3', reverse: 5'-GGAGTTGAGTATGTCCGAGT-3'; PGC-1α: forward: 5'-GTGCAGCCAAGACTCTGTATGG-3', reverse: 5'-GTCCAGGTCATTCACATCAAGTTC-3'; TFAM: forward: 5'-GGTGTATGAAGCGGATTT-3', reverse: 5'-CTTTCTTCTTTAGGCGTTT-3'; GAPDH: forward: 5'-AAGGTGAAGGTCGGAGTCAA-3', reverse: 5'-AATGAAGGGGTCATTGATGG-3'. Samples were tested in triplicates and data from five independent experiments were used for analysis.

### 4.15. Western Blot Analysis

Equivalent amounts of protein (40 μg per lane) were loaded and separated by 10% sodium dodecyl sulfate (SDS)-PAGE gels, and transferred to polyvinylidene difluoride (PVDF) membranes (Invitrogen, Carlsbad, CA, USA). The membranes were blocked with 5% nonfat milk solution in tris-buffered saline with 0.1% Triton X-100 (TBST, Invitrogen, Carlsbad, CA, USA) for 1 h, and then incubated overnight at 4 °C with the primary Sirt3 antibody (1:1000) or β-actin (1:600) antibody dilutions in TBST. After that the membranes were washed and incubated with secondary antibody for 1 h at room temperature. Immunoreactivity was detected with Super Signal West Pico Chemiluminescent Substrate (Thermo Scientific, Rockford, IL, USA). An analysis software named Image J (National Institutes of Health, Boston, MA, USA) was used to quantify the optical density of each band.

### 4.16. Statistical Analysis

Statistical analysis was performed using SPSS 16.0 (SPSS Inc., Chicago, IL, USA), a statistical software package. Statistical evaluation of the data was performed by one-way analysis of variance (ANOVA) followed by Bonferroni’s multiple comparisons. A value of *p* < 0.05 was considered statistically significant.

## 5. Conclusions

In conclusion, our results showed that the expression of Sirt3 was significantly increased at both mRNA and protein levels after H_2_O_2_ treatment in primary cultured cortical neurons. Down-regulation of Sirt3 using specific targeted siRNA exacerbated the H_2_O_2_-induced neuronal injury, whereas overexpression of Sirt3 exerted protective effects through attenuating ROS generation and activation of endogenous antioxidant enzymes. The increased expression of Sirt3 induced by oxidative stress might be an endogenous protective mechanism, which is partly dependent on the preservation of mitochondrial calcium homeostasis and enhancement of mitochondrial biogenesis. Thus, metabolic rescue observed upon overexpression of Sirt3 may represent an appropriate strategy to avoid neuronal death in a broad range of oxidative stress related brain disorders.
